# Utilizing a naturopathic mouthwash with selective antimicrobial effects against multispecies oral biofilms for prevention of dysbiosis

**DOI:** 10.3389/froh.2025.1529061

**Published:** 2025-05-19

**Authors:** Danyal A. Siddiqui, Yi-Wen C. Tsai, Juliana Giron Bastidas, Marzieh S. Jazaeri, Georgios A. Kotsakis

**Affiliations:** ^1^ Department of Oral Biology, Rutgers School of Dental Medicine, Newark, NJ, United States; ^2^ Clinical Research Center, Rutgers School of Dental Medicine, Newark, NJ, United States; ^3^ Translational Periodontal Research Lab, Department of Periodontics, University of Texas Health Science Center at San Antonio, San Antonio, TX, United States; ^4^ Department of Periodontology, School of Dentistry, Tri-Service General Hospital and National Defense Medical Center, Taipei, Taiwan

**Keywords:** oral rinses, chlorhexidine, antimicrobial(s), oral biofilms, periodontitis, gingivitis

## Abstract

**Introduction:**

Oral rinses intended for the prevention and treatment of periodontal diseases have traditionally focused on bactericidal effects. This study evaluates the efficacy of a naturopathic mouthwash containing plant attenuations and propolis against common gram- pathogenic and gram+ commensal oral species in comparison to conventional antiseptic oral rinses.

**Methods:**

*Streptoccoccus oralis, Streptococcus gordonii, Veillonella parvula, Fusobacterium nucleatum, and Porphyromonas gingivalis* were cultured and treated with naturopathic StellaLife® VEGA® Oral Rinse (SL), 0.12% chlorhexidine gluconate (CHX), LISTERINE® COOL MINT® mouthwash (LIS), or phosphate-buffered saline (PBS) as negative control. Firstly, planktonic bacterial growth was assessed through optical density measurements and colony-forming unit (CFU) counts. Subsequently, a 4-species or clinical ex vivo multispecies biofilm was used to evaluate antibiofilm effects through selective agar plating and fluorescence in situ hybridization (FISH) or live-dead biofilm imaging, respectively. Lastly, cytocompatibility to oral rinses was tested using a 3D human fibroblast spheroid model.

**Results:**

SL significantly inhibited the growth of disease-associated *F. nucleatum and P. gingivalis* 12 and 120 h, respectively, after treatment, while exhibiting lower toxicity toward commensal *S. oralis, S. gordonii*, and *V. parvula* vs. *LIS* or CHX (all *p* < 0.05). Correspondingly, in 4-species biofilms, selective agar plating and FISH-staining showed decreased abundance of *F. nucleatum and P. gingivalis* after 4 h recovery following SL treatment vs. PBS control while maintaining a robust commensal biofilm of *S. oralis and V. parvula*. In contrast, CHX or LIS treatment demonstrated non-selective killing, leading to sparse biofilms with residual *F. nucleatum and P. gingivalis*. When tested against clinical ex vivo multispecies biofilms, all oral rinses showed significant antibiofilm effects (all *p* < 0.001), disrupting biofilm structure and reducing bacterial viability. Lastly, 3D human fibroblast spheroids treated with CHX or LIS displayed greater cytotoxicity with detachment of cellular debris from the spheroid mass, while spheroids exposed to SL exhibited minimal cell death with cellular viability maintained across the spheroid structure.

**Discussion:**

The SL homeopathic rinse demonstrated selective action on oral bacteria, preferentially reducing pathogen bacterial load while preserving commensal species with high cytocompatibility. Future validation in human studies is needed to assess its selective antimicrobial activity to maintain a eubiotic oral microbiome and explore broader applications in oral health.

## 1 Introduction

The use of oral rinses (also called mouthrinses/mouthwashes) has significantly increased in the last two decades with about 120 million individuals in the US using mouthwash daily and over two-thirds using it for oral treatment (1). The indications range from pre- and post-dental procedures for oral bacterial control and healing to adjunctive treatment of periodontal diseases and long-term maintenance as part of oral hygiene. However, mouthwashes have also been scrutinized due to their negative effect on protective commensal microbes (i.e., “good bacteria”) that facilitate tissue health and homeostasis (2). In fact, the use of antimicrobial rinses with non-selective cytotoxicity may promote oral microbial dysbiosis, defined by loss of commensal bacteria and an increase in pathogenic microbiota, leading to elevated host tissue inflammation (2). Furthermore, the impact of mouthwash-related depletion of oral commensal species extends beyond oral dysbiosis and includes adverse systemic health effects such as cardiovascular disease (hypertension) and prediabetes/diabetes (2–4). This underlines the need for novel, targeted mouthwash formulations that exhibit selective cytotoxicity rather than a “gunshot” approach to balance oral microbial communities instead of eradicating them (2).

Various plant-based attenuations and dilutions have demonstrated antimicrobial efficacy against periodontal pathogens. Mistry et al. assessed *Azadirachta indica* (neem), *Ocimum sanctum* (tulsi), *Mimusops elelngi* (bakul), *Tinospora cardifolia* (giloy) and chlorhexidine gluconate on common endodontic pathogens like *Streptococcus mutans*, *Enterococcus faecalis* and *Staphylococcus aureus* (5, 6). Methanolic extract of *A. indica*, *O. sanctum*, *M. elengi*, and *T. cardifolia* showed substantial antimicrobial activity against *S. mutans*, *E. faecalis* and *S. aureus* similar to that of chlorhexidine gluconate (6). Several independent investigations have shown significant antimicrobial and antiviral effects of neem and *Calendula officinalis* as a potential anti-gingivitis agent (6–15). Additionally, *Eichenesia* exhibited antifungal properties and exerted positive effects on the immune system (16–19). Lastly, propolis, a resin-like mixture comprised of beeswax and tree buds exudates, showed immunomodulatory activity and reduced dental plaque formation while being non-toxic to gingival fibroblasts (20–23).

Recently, a naturopathic mouthwash based on these homeopathic attenuations and propolis has been introduced and found to promote gingival wound healing on the transcriptional and translational levels (24, 25). Moreover, it had virtually no cytotoxicity to host tissue oral cells as compared to existing antimicrobial rinses like chlorhexidine, which are generally cytotoxic to host tissues and may delay wound healing (24, 25). However, there is limited information on the antimicrobial effects of this mouthwash. Therefore, the goal of this study was to evaluate the anti-bacterial and anti-biofilm effects of the naturopathic oral rinse vs. commonly used antiseptic rinses.

## 2 Methodology

The general methodology for this experimental work included four levels of assays. Firstly, a higher throughput screening was conducted on broth cultures of individual oral species to determine whether the inhibitory or bactericidal effects of the naturopathic mouthwash were selective towards specific oral taxa. Next, a four-species model consisting of commensal *S. oralis* and *V. parvula* and pathogens *F. nucleatum* and *P. gingivalis* was utilized to evaluate any selective bactericidal effects of the mouthwash against each species in nascent, adherent biofilm. Subsequently, an established *ex vivo* oral biofilm model (26, 27) was employed to assess the anti-biofilm effects of the naturopathic mouthwash as compared to commercially available mouthwashes for translational impact. This *ex vivo* model has been previously validated and includes over 80 different taxa of oral commensal and pathogenic bacteria, including taxa challenging to retain in culture such as *Treponema* (26, 27). Lastly, a translational 3D spheroid fibroblast culture was used to demonstrate the impact of oral rinse treatment on host soft tissue cytocompatibility.

### 2.1 Bacterial strains and culture conditions

The following oral bacterial species were evaluated: *Streptoccoccus oralis* (ATCC 35037), *Streptococcus gordonii* DL1, *Veillonella parvula* PK1910, *Fusobacterium nucleatum* (ATCC 25586), and *Porphyromonas gingivalis* (ATCC 33277). All bacteria were cultivated anaerobically (80% N_2_, 10% CO_2_ and 10% H_2_) at 37°C. *S. oralis* and *S. gordonii* were struck from −80°C stocks onto Brain Heart Infusion (BHI) agar (BD Bacto) plates while *V. parvula* was struck onto BHI agar plates supplemented with 0.6% (v/v) sodium DL-lactate (BHIL). *F. nucleatum* was struck onto Brucella agar containing 10 mg/L hemin and 10 mg/L vitamin K1 (HiMedia Laboratories) and supplemented with 5% defibrinated sheep blood (Hardy Diagnostics). Agar plates were incubated for 48–72 h prior to inoculating single colonies of *S. oralis*, *S. gordonii*, and *F. nucleatum* in 5 ml of BHI broth supplemented with 0.1 µg/ml vitamin K1 and 5 µg/ml hemin. *V. parvula* was inoculated in BHIL broth. *P. gingivalis* was directly inoculated (50 µl) from frozen stocks into 5 ml of supplemented BHI broth. All bacteria were grown until late log or early stationary phase was reached based on optical density measurements at 600 nm wavelength (OD_600_) before treatment. Culturing of all strains was performed under BSL2 conditions and was approved by an institutional biosafety committee. Additionally, this study incorporated a clinically isolated multispecies sample of periodontitis, comprising a diverse range of over thirty bacterial genera to enhance clinical relevance and explore the extent of antibiofilm effects, including fastidious anaerobes like *Porphoromonas*, *Bacteroides*, and *Tannerella* and other periodontal pathogens like *Fusobacterium*, *Prevotella*, and *Campylobacter* (Supplementary Table S1). Detailed isolation, composition, retention of bacterial diversity, and culture methods for the *ex vivo* ecological biofilm have been previously described (26, 27).

### 2.2 Assessment of oral rinsing treatment on planktonic and adherent bacterial growth

The following oral rinses were studied to evaluate their efficacy against neutralizing planktonic oral bacterial growth: 3M™ Peridex™ Chlorhexidine Gluconate 0.12% Oral Rinse (CHX), LISTERINE® COOL MINT® mouthwash (LIS), and StellaLife® VEGA® Oral Rinse (SL). Phosphate buffered saline (1× PBS) served as a negative control for oral rinsing treatment. Based on OD_600_ readings (using standard curves relating known OD_600_ values with viable CFU counts; data not shown), 5 × 10^8^ CFU/ml of all monocultures and multispecies bacteria were collected and pelleted by centrifugation at 13,000 × g for 4 min. The supernatant was discarded, and bacterial pellets were resuspended in 100 µl of oral rinse for 1 min by vigorous pipetting. Afterward, bacterial resuspension in oral rinse was diluted 100-fold in PBS immediately and further diluted 100-fold by inoculating 30 µl of PBS-diluted culture in 3 ml of respective growth medium. Bacterial growth post rinsing treatment was monitored periodically via OD_600_ readings and by plating 10-fold serial dilutions of bacterial medium in respective growth medium agar (*n* = 4 biological replicates). Based on each bacterial species growth times, *S. oralis* and *S. gordonii* growth were evaluated after 0, 8, 12, 18, and 24 h post-treatment while *V. parvula* and *F. nucleatum* were evaluated after 0, 12, 18, 24, and 36 h. *P. gingivalis* was evaluated after 0, 48, 96, 120, and 168 h, and multispecies bacteria were evaluated after 0, 12, 24, 48, and 96 h.

To assess bactericidal killing of the oral rinses against adherent bacteria, a four-species model comprised of *S. oralis*, *V. parvula, F. nucleatum*, and *P. gingivalis* was employed (*n* = 4 biological replicates). Four-species growth medium consisted of BHI broth supplemented with 5 μg/mL hemin, 0.1 μg/mL vitamin K1, and 0.6% (v/v) sodium DL-lactate. Single species cultures were grown as previously described and combined in equal CFU proportions (1:1:1:1) based on OD_600_ readings for a total concentration of 10^8^ CFU/ml. One ml of 4-species culture was seeded per 12-well and incubated for 1 h anaerobically at 37°C to facilitate coaggregation and attachment. The medium with loosely bound bacteria was removed and treated with 1 ml of oral rinse for 1 min and immediately rinsed twice with PBS. Fresh supplemented BHI broth was added, and bacterial recovery was allowed to proceed for 4 h anaerobically at 37°C. Adherent bacteria in early biofilm was harvested by vigorous pipetting. Serial dilutions of treated biofilms were plated on the following selective agar to enumerate CFU count: tryptic soy blood agar (TSBA) supplemented with 5% defibrinated sheep blood (Colorado Serum Company) with 8 mg/L vancomycin hydrochloride and 1 mg/L oxacillin sodium salt for *V. parvula*, TSBA with 4 mg/L vancomycin hydrochloride, 1 mg/L erythromycin, and 1 mg/L norfloxacin for *F. nucleatum*, and TSBA with 1 g/L phosphomycin for *P. gingivalis*.

### 2.3 Analysis of bactericidal effects on *in vitro* multispecies biofilm composition

To investigate the bactericidal effect of the oral rinses on adherent biofilms, 4-species biofilm were prepared as described in Section 2.2 and seeded in 35-mm glass coverslip-bottom dishes for analysis of qualitative assessment of abundance and spatial organization of biofilm samples using fluorescence *in situ* hybridization (FISH) with species-specific probes. After treatment and 4 h recovery, the biofilm samples were rinsed once with PBS and fixed with 4% paraformaldehyde (PFA) for 3 h at ambient temperature. Biofilms were rinsed twice with deionized water, permeabilized with 2 mg/ml lysozyme in 10 mM Tris hydrochloride (Tris HCl) for 9 min at 37°C, dehydrated in 50%, 80%, and 90% ethanol for 5 min, dried for 10 min, and stored overnight at 4°C. The following day, biofilm samples were incubated for 30 min at 37°C in 0.2 ml of hybridization buffer consisting of 900 mM sodium chloride (NaCl), 20 mM Tris HCl, 30% (v/v) formamide, and 0.01% sodium dodecyl sulfate (SDS) in deionized water. One µl of the following FISH probes (1,000 ng/ml stock) were then added to the hybridization buffer at a final concentration of 5 ng/ml to stain biofilm samples and incubated for 3 h at 42°C: STR405 conjugated to Pacific Blue dye (5'-TAGCCGTCCCTTTCTGGT-PB) for *S. oralis*, FUS714 conjugated to Alexa Fluor 488 dye (5'-GGCTTCCCCATCGGCATT-AF488) for *F. nucleatum*, PGI1160 conjugated to Alexa Fluor 594 dye (5'-CCTCACGCCTTACGACGG-AF594) for *P. gingivalis*, and VEI488 conjugated to Alexa Fluor 647 dye (5'-CCGTGGCTTTCTATTCCG-AF647) for *V. parvula*. Hybridization buffer was removed, and samples were rinsed twice by incubating for 15 min at 37°C in 1 ml wash buffer consisting of 900 mM NaCl, 20 mM Tris HCl, 5 mM ethylenediaminetetraacetic acid (EDTA), and 0.01% (v/v) SDS. Biofilm samples were then mounted with 10 µl of SlowFade Diamond Antifade Mountant (Invitrogen) and glass coverslips. Fluorescent imaging of biofilm samples was performed at 100× magnification using a Leica DMi8 inverted fluorescence microscope equipped with Leica THUNDER Imaging Systems. 3D scans of fluorescent biofilms were processed using Large Volume Computational Clearing (LVCC) and transforming to 2D images using maximum projection.

### 2.4 Evaluation of bactericidal effects on clinical *ex vivo* multispecies biofilms

To study bactericidal effects, *ex vivo* multispecies biofilms were grown in modified SHI medium in this study as previously described (26). Briefly, −80°C frozen stock samples were thawed and inoculated in SHI broth. After 24 h of growth under anaerobic condition at 37°C, 1 ml aliquots of multispecies culture diluted to 5 × 10^7^ CFU/ml were added into sterile 24-well plates and incubated for 24 h. Subsequently, multispecies biofilms were washed with sterile deionized water and then stimulated with 1 ml of CHX, LIS, SL or PBS as a negative control (*n* = 3 biological replicates). 70% isopropyl alcohol (IPA) was used as a positive control for antibiofilm assessment per established disinfection guidelines (28). After stimulation, the treated biofilms were washed with deionized water to remove residual solutions and stained with 10 µM SYTO® 9 green fluorescent nucleic acid stain and 60 µM propidium iodide (Filmtracer™ LIVE/DEAD™ Biofilm Viability Kit) according to manufacturer's instructions. After staining, biofilms were gently washed once with deionized water to remove residual dye. Fluorescent imaging was performed at 10× and 20× magnification with an Invitrogen™ EVOS™ M5000 Imaging System. 3D reconstruction and analysis were performed using ImageJ2 (Fiji, v 2.14.0/1.54f), and the area coverage in separated green (live) and red (dead) fluorescent channels was measured. The percentage of area coverage for each channel was calculated by dividing the live or dead area coverage by the sum of both areas.

### 2.5 Impact of oral rinsing treatment on host soft tissue cytocompatibility

Human gingival fibroblast (HGF-1) cell line (ATCC CRL-2014) was cultured in Dulbecco's Modified Eagle Medium (DMEM) supplemented with 10% fetal bovine serum (FBS) at 37°C and 5% CO_2_. To form 3D spheroids of fibroblasts, agarose micro-molds were prepared using commercially available polydimethylsiloxane (PDMS) molds (Sigma-Aldrich) with a microsphere pattern. Type I agarose was dissolved in PBS to a final concentration of 3.5%, melted by microwave heating, and poured into the molds. After cooling to ambient temperature, solid micro-molds were harvested and incubated for 2 h in growth medium. Next, the culture medium was removed, and 6 × 10^4^ fibroblasts were seeded into each micro-mold in 200 µl of culture medium within 12-well plates. After 2 h incubation to allow settling of fibroblasts into the micro-mold wells by gravity, 1 ml of medium was added to fully submerge the micro-molds in medium. Fibroblast spheroid cultures were maintained in standard growth conditions for 7 days.

To investigate the effect of oral rinse treatment on 3D fibroblast culture viability, spheroids were treated for 1 min with the oral rinses CHX, LIS, or SL. A 1% (v/v) Triton X-100 solution or PBS were used as positive and negative controls for cell death, respectively. A Live/Dead assay (Invitrogen™, Ref. L3224) was performed to assess cell viability, following the manufacturer's protocol (*n* = 3 biological replicates). Fluorescence images were acquired using the Leica DMi8 inverted fluorescence microscope.

### 2.6 Statistical analysis

A standard two-way ANOVA test was used for statistical analyses in GraphPad Prism 8.4.1., and significance was determined at a *p*-value of ≤0.05 followed by *post-hoc* testing. When multiple timepoints were assessed, repeated measures ANOVA models were implemented for each condition and timepoints followed by appropriate *post hoc* tests.

## 3 Results

### 3.1 Antibacterial effects on planktonic growth

**Figure 1:**
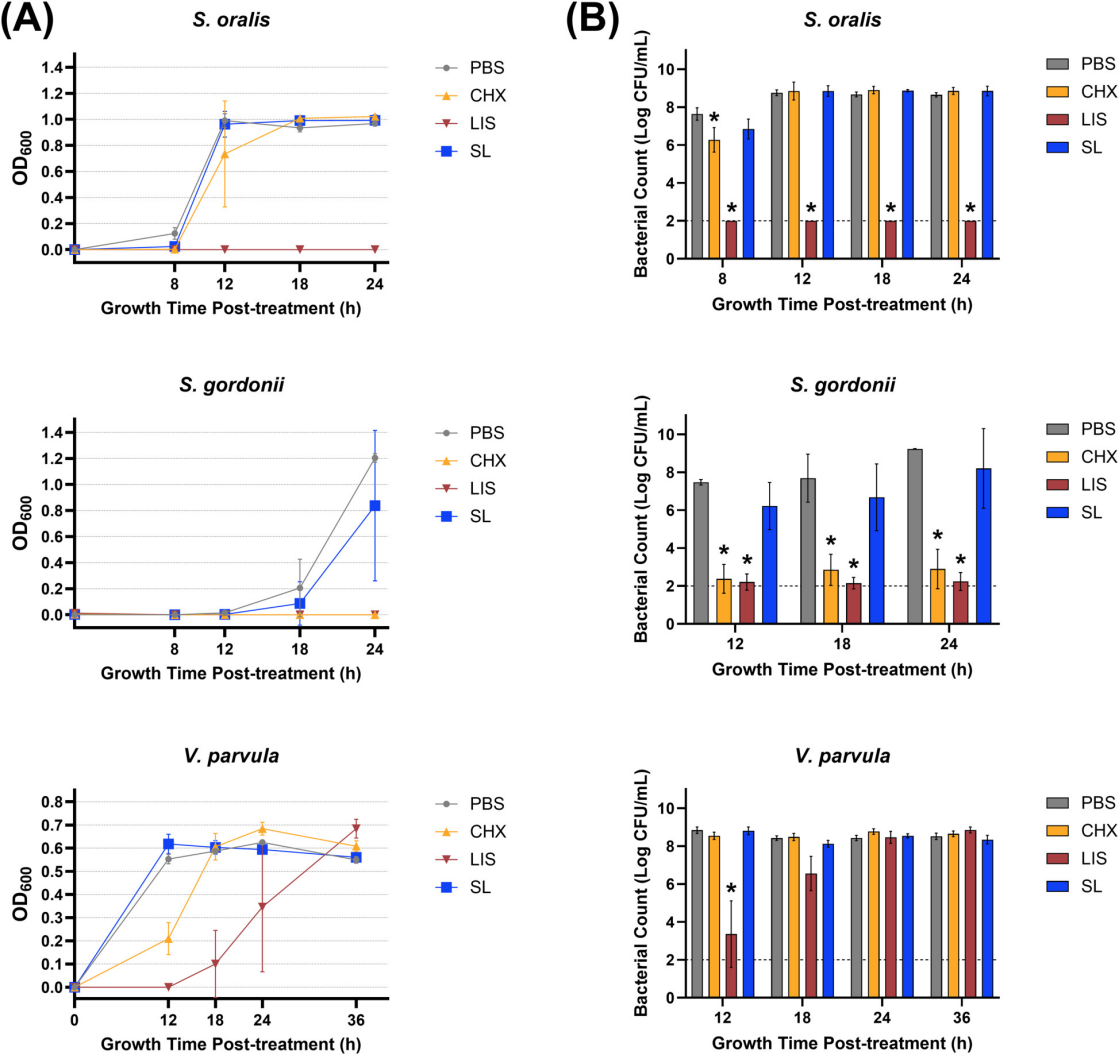
**(A)** Growth curves measured by optical density at 600 nm wavelength (OD600) and **(B)** viable bacterial colony-forming unit (CFU) counts of *S. oralis*, *S. gordonii*, and *V. parvula* monocultures at various time points after treatment with phosphate buffered saline (PBS), 0.12% chlorhexidine gluconate (CHX), LISTERINE® mouthwash (LIS), or StellaLife® VEGA® Oral Rinse (SL). Statistical comparisons of antibacterial effect of each treatment against PBS-treated control group (*p* ≤ 0.05 followed by post-hoc testing).

Planktonic bacteria were treated with an herbal StellaLife® (SL) oral rinse or conventional chlorhexidine (CHX) or LISTERINE® (LIS) mouthwashes to assess comparative antibacterial effects. Bacteria treated with sterile phosphate-buffered saline (PBS) served as negative control. Figure 1A presents the growth curves of the treated early colonizing commensal species (*S. oralis*, *S. gordonii*, *V. parvula*), as measured by optical density at 600 nm wavelength (OD_600_), at different time points, with statistical comparisons made against the PBS-treated control group (Supplementary Table S2). SL did not inhibit any commensal bacterial growth as compared to PBS. In contrast, CHX significantly impeded the growth of *S. oralis* and *V. parvula* up to 12 h post-treatment and stunted the growth of *S. gordonii*, which did not recover even after 24 h (*p* < 0.0001). Similarly, LIS eliminated *S. oralis* below the limit of detection up to 24 h after treatment, stunted the growth of *S. gordonii* after 24 h, and impeded *V. parvula* growth until recovery after 18 h.

Figure 1B depicts viable bacterial counts [as the logarithm of colony-forming unit (CFU) per ml] corresponding to OD_600_ readings at different time points after treatment. These results align closely with the optical density data, demonstrating that SL generally exhibited lower toxicity toward commensal *S. oralis*, *S. gordonii*, and *V. parvula* as compared to LIS or CHX. SL only marginally inhibited *S. oralis* counts by ∼10-fold 8 h after treatment vs. PBS control, with recovery observed at the 12-h time point (Figure 1B). On the other hand, LIS severely reduced *S. oralis* and *S. gordonii* counts (∼1,000,000-fold decrease vs. PBS) at all time points after treatment. Likewise, LIS lowered *V. parvula* counts as compared to PBS until 24 h after treatment. CHX significantly reduced the growth of *S. oralis* by ∼100-fold vs. PBS after 8 h and to a greater extent for *S. gordonii* (∼1,000,000-fold decrease) at all time points but did not affect *V. parvula* counts.

**Figure 2:**
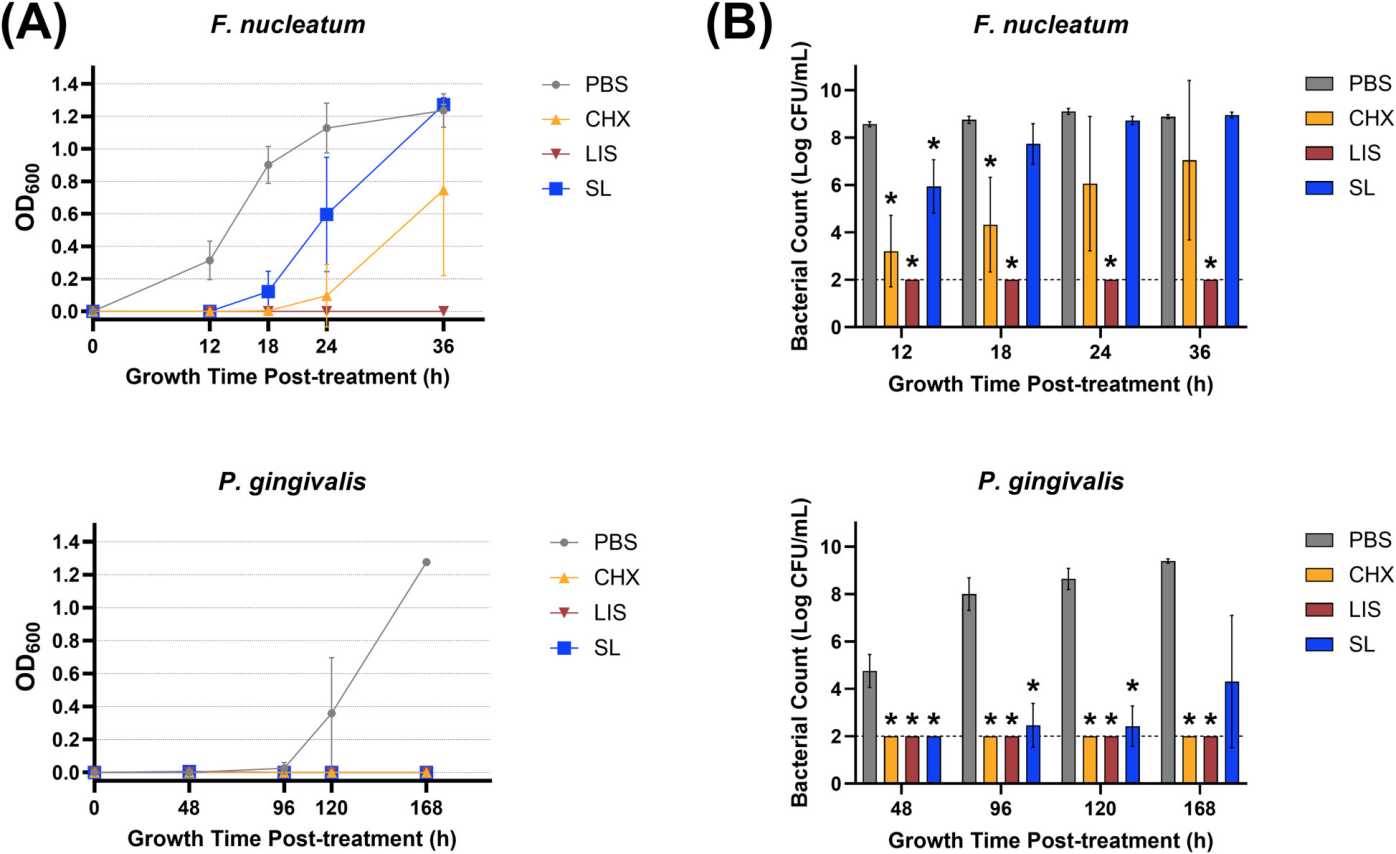
**(A)** Growth curves measured by optical density at 600 nm wavelength (OD600) and **(B)** viable bacterial colony-forming unit (CFU) counts of *F. nucleatum* and *P. gingivalis* monocultures at various time points after treatment with phosphate buffered saline (PBS), 0.12% chlorhexidine gluconate (CHX), LISTERINE® mouthwash (LIS), or StellaLife® VEGA® Oral Rinse (SL). Statistical comparisons of antibacterial effect of each treatment against PBS-treated control group (*p* ≤ 0.05 followed by *post-hoc* testing).

Figure 2A depicts the effect of oral rinse treatment based on the OD600 reading of late colonizing pathogens (*F. nucleatum*, *P. gingivalis*). When applied to pathogenic bacteria, SL significantly suppressed growth up to 12 h after treatment for *F. nucleatum* and 96 h for *P. gingivalis*, similar to CHX and LIS. However, CHX maintained greater suppression of *F. nucleatum* up to 36 h post-treatment, and both CHX and LIS eliminated *P. gingivalis* growth below the limit of detection up to 168 h after treatment. When treating the more complex multispecies composition, SL had a total microbial growth profile closely resembling PBS control while CHX retarded growth up to 96 h after treatment and LIS eliminated all cultivable species below the limit of detection.

Regarding pathogenic bacterial load shown in Figure 2B, SL significantly decreased *F. nucleatum* counts by ∼100-fold vs. PBS after 12 h and *P. gingivalis* counts by up to ∼1,000,000-fold at all timepoints. LIS maintained *F. nucleatum* and *P. gingivalis* counts below the limit of detection at all time points after treatment. CHX also significantly suppressed *F. nucleatum* and *P. gingivalis* counts by up to ∼1,000,000-fold after 24 h and 168 h, respectively, after treatment.

### 3.2 Bactericidal effects on *in vitro* multispecies biofilm

**Figure 3:**
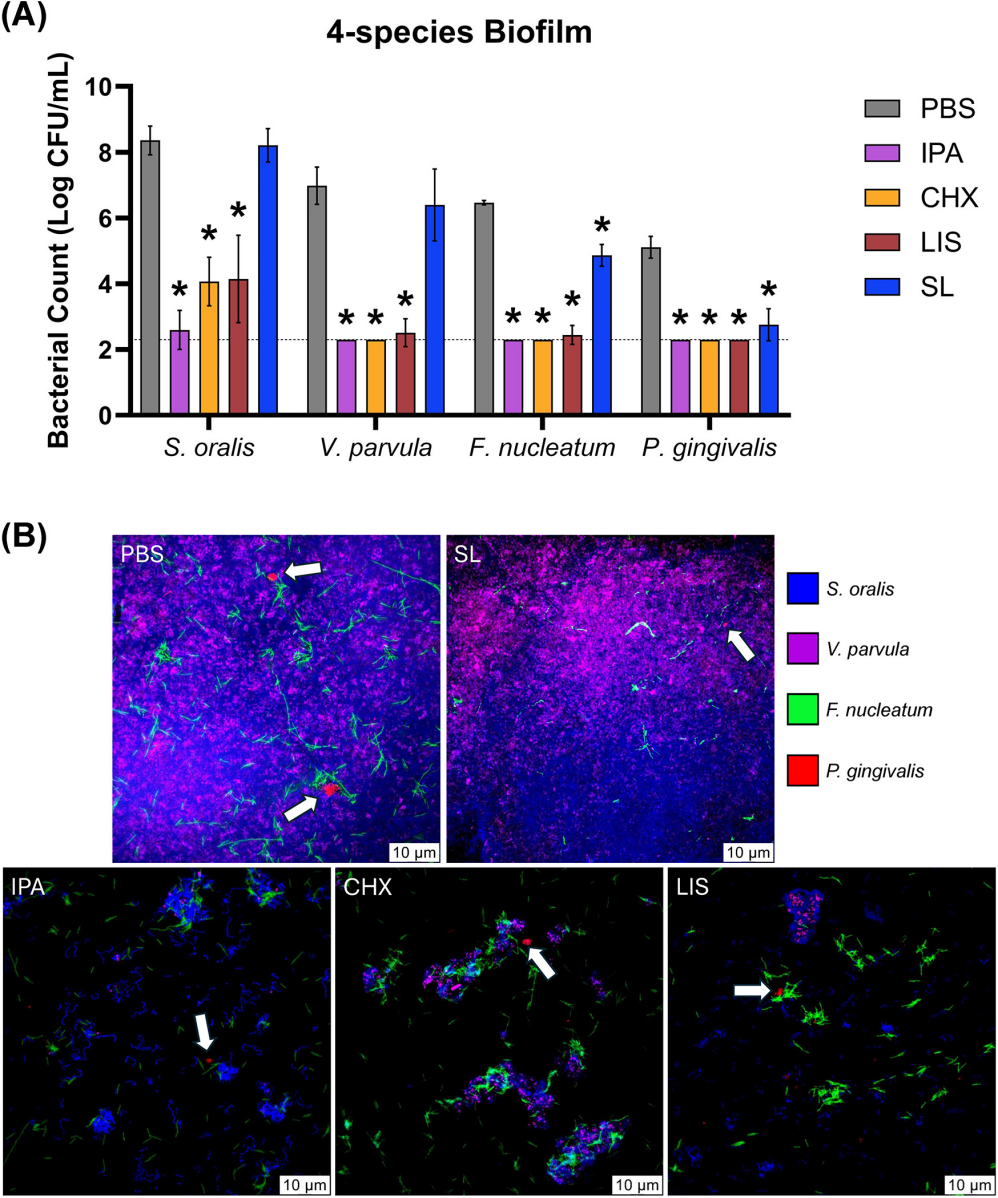
**(A)** Viable bacterial colony-forming unit (CFU) counts and **(B)** stained fluorescent in situ hybridization (FISH) images of *S. oralis* (blue), *V. parvula* (purple), F. nucleatum (green) and *P. gingivalis* (red) bacterial cells in four-species culture after 4 h growth recovery following treatment with phosphate buffered saline (PBS), 0.12% chlorhexidine gluconate (CHX), LISTERINE® mouthwash (LIS), or StellaLife® VEGA® Oral Rinse (SL). Statistical comparisons of antibacterial effect of each treatment against PBS-treated control group (*p* ≤ 0.05 followed by *post-hoc* testing) within each bacteria group. White arrows denote *P. gingivalis* colonies (red-fluorescently stained) in four-species biofilm.

In Figure 3A, the bactericidal effect of the oral rinses against bacterial biofilm was assessed using a 4-species culture of *S. oralis*, *V. parvula*, *F. nucleatum*, and *P. gingivalis*. Selective agar plating of treated biofilms revealed that SL significantly reduced CFU counts of *F. nucleatum* and *P. gingivalis* by ∼100-fold and ∼1,000-fold, respectively, (*p* < 0.05) while maintaining commensal *S. oralis* and *V. parvula* CFU counts comparable to PBS-treated control. In contrast, CHX and LIS significantly reduced CFU counts for both commensal and pathogenic species (*p* < 0.05). In Figure 3B, representative images of FISH-stained biofilms showed that SL treatment decreased the apparent abundance of *F. nucleatum* and *P. gingivalis* (white arrows) in nascent biofilm after 4 h vs. PBS control while maintaining a robust commensal biofilm comprised of *S. oralis* and *V. parvula*. In contrast, CHX and LIS severely mitigated recovery growth of both commensal and pathogens, resulting in sparse biofilm formation with low abundance of all species similar to IPA-treated control.

### 3.3 Bactericidal effects on clinical *ex vivo* multispecies biofilm

**Figure 4:**
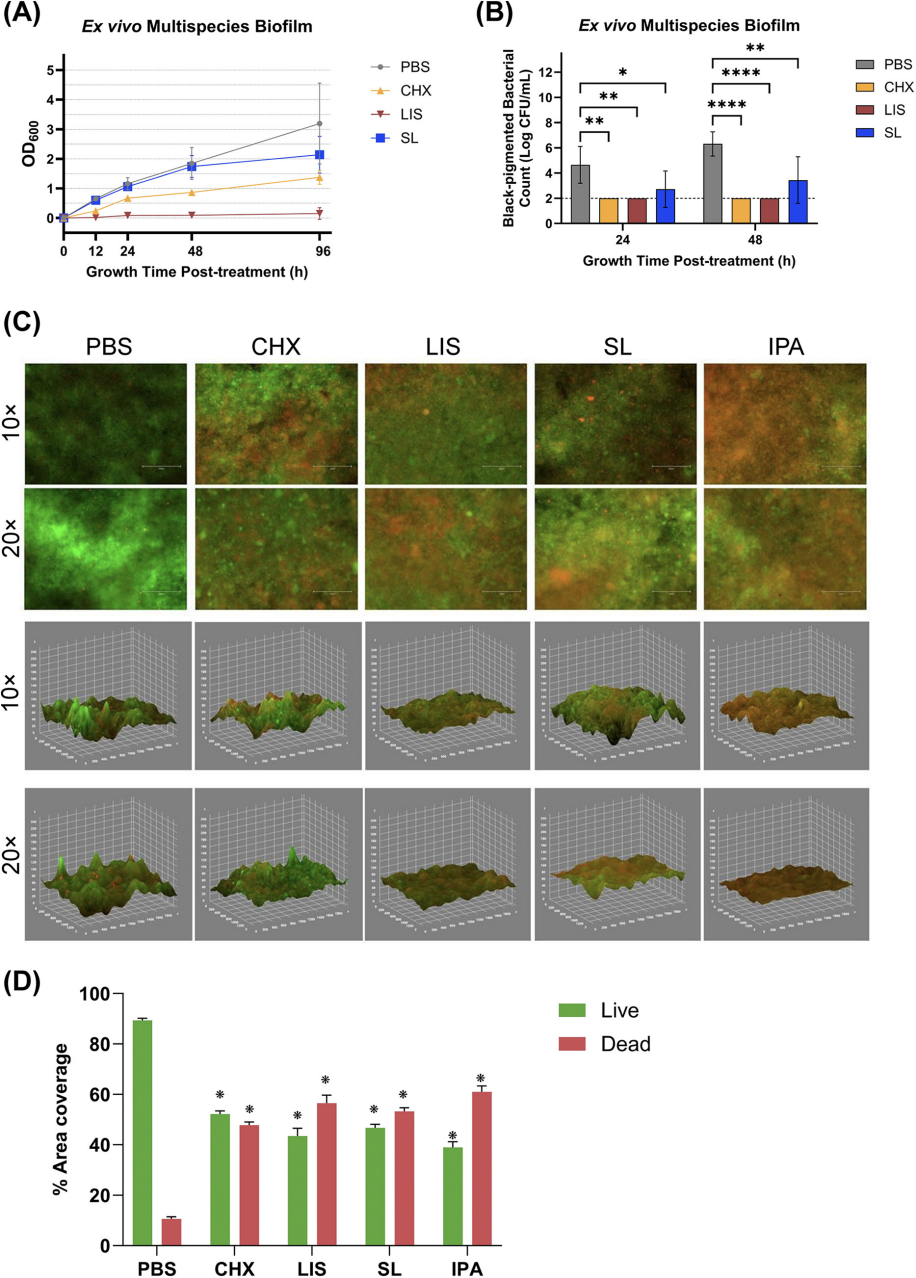
**(A)** Growth curves measured by optical density at 600 nm wavelength (OD600) and **(B)** viable, black-pigmented bacterial colony-forming unit (CFU) counts of planktonic *ex vivo* multispecies cultures up to 96 h after treatment with phosphate buffered saline (PBS), 0.12% chlorhexidine gluconate (CHX), LISTERINE® mouthwash (LIS), or StellaLife® VEGA® Oral Rinse (SL). Statistical comparisons of antibacterial effect of each treatment against PBS-treated control group (*p* ≤ 0.05 followed by post-hoc testing). **(C)** Live/dead staining of 24 h multispecies biofilm grown from clinical ex vivo samples after treatment with PBS, CHX, LIS, SL, or 70% isopropyl alcohol (IPA) as 2D projections (top panel) or 3D reconstruction (bottom panel) of the multispecies biofilm depicting disruption of biofilm spatial structure and reduction of residual bacteria following treatment. **(D)** Percentage of live or dead area coverage of the multispecies biofilm calculated by dividing the percentage of area coverage of the green (live) or red (dead) fluorescence by the sum of both areas.

The bactericidal effect of oral rinses against ex vivo multispecies culture after oral rinse treatment is shown in Figure 4. Interestingly, only SL treatment maintained a recovery growth curve profile for planktonic multispecies culture similar to PBS control up to 48 h while CHX and LIS stunted or eliminated growth at all time points, respectively (Figure 4A). The total and black-pigmented CFU counts were recorded for further analysis, as black-pigmented colonies typically represent more periodontopathic species in the oral cavity. Figure 4B depicts the black-pigmented CFU counts 24 and 48 h treatment. Average black-pigmented CFU counts increased by 48 h for the PBS control group but were maintained at significantly lower counts after treatment by all oral rinses (all *post-hoc p* < 0.05) in descending order of LIS = CHX > SL.

To assess the surface coverage and viability of a robust, clinical *ex vivo* multispecies biofilm after oral rinse treatment, fluorescence microscopy was employed on stained biofilms. Figure 4C (top panel) displays the 2D projection of biofilms 24 h after treatment, where live and dead bacteria appear as green or red, respectively, after staining with SYTO® 9 and propidium iodide. Semi-quantitative assessment of the fluorescent images in Figure 4D demonstrated that all oral rinses had a significant reduction in viable bacteria as compared to PBS-treated control (all *p* < 0.001). Inter-group differences among oral rinsing treatments revealed that LIS had the greatest reduction in green vs. red fluorescence with an average live/dead ratio of 44.31 ± 2.5% and 55.68 ± 2.5%, respectively. However, these differences were not significant among groups or towards the isopropanol (IPA) positive control for antibacterial-induced cell death (*p* > 0.05).

As can be seen in the 2D images in Figure 4C (top panel), the PBS-treated group (negative control for antibiofilm treatment) exhibited a higher coverage of live bacteria, while IPA-treated group (positive control for treatment) showed a higher coverage of dead bacteria. In comparison, biofilms treated with CHX, LIS, or SL displayed relatively balanced ratio of live to dead bacteria. Notably, the LIS-treated group seemed to have a higher ratio of dead bacteria.

Additionally, Figure 4C (bottom panel) provides 3D reconstructions generated from the corresponding merged fluorescent channels. These reconstructions show the disruption of biofilm spatial structure and reduction in biofilm volume following treatment. PBS and CHX appear to have a higher presence of green fluorescence in addition to more steeper peaks and deeper valleys, indicating that the biofilms remained intact. In contrast, biofilms treated with IPA, LIS, or SL appeared to have more red fluorescence with shallower 3D surface features consistent with bacterial death and biofilm reduction.

### 3.4 Impact of oral rinse on host soft tissue cytocompatibility

**Figure 5:**
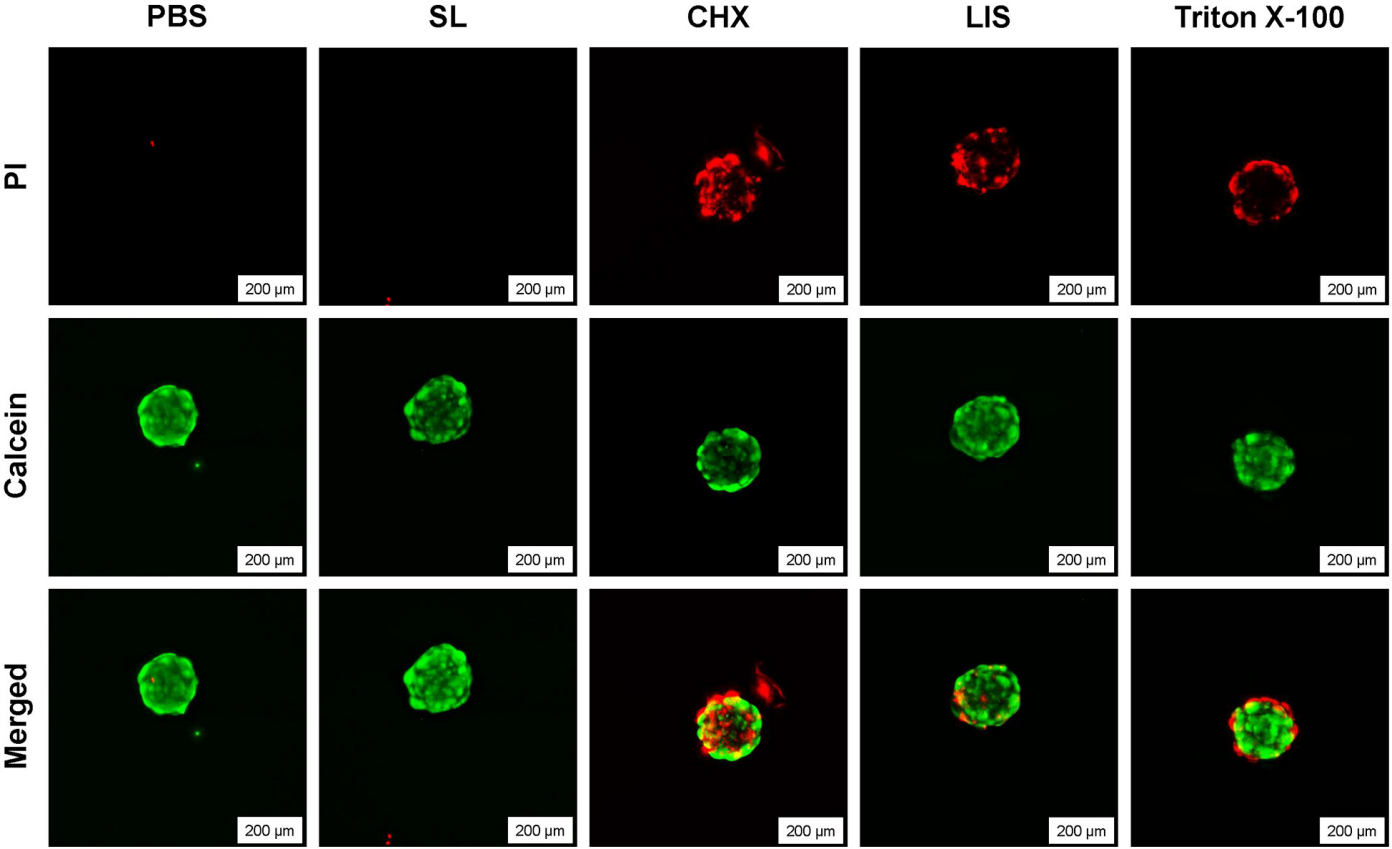
Live/dead staining of 3D human fibroblast spheroids after treatment with phosphate buffered saline (PBS), 0.12% chlorhexidine gluconate (CHX), LISTERINE® mouthwash (LIS), or StellaLife® VEGA® Oral Rinse (SL), or 1% Triton X-100 at 20× magnification depicting cytocompatibility after treatment with PBS or SL and loss of viability after treatment with CHX, LIS, or Triton X-100.

Figure 5 demonstrates the effect of exposure of oral rinsing treatments on the viability of 3D fibroblast spheroids based on live-dead staining and fluorescence imaging. Spheroids treated with CHX displayed substantial cytotoxicity, characterized by widespread cell death throughout the entire spheroid, including the central core, along with evident detachment of cellular debris from the spheroid mass. In comparison, spheroids exposed to LIS exhibited moderate cell death, mainly restricted to the outer layers. Similarly, the positive control (1% Triton X-100) induced peripheral cell death, likely due to the limited diffusion of the agent within the dense spheroid structure. In contrast, the untreated control and SL-treated spheroids showed minimal cytotoxicity (red fluorescence), indicating the presence of viable cells across the entire spheroid.

## 4 Discussion

The present study investigated SL, a novel naturopathic mouthwash, against a range of oral bacteria encompassing periodontal pathogens and commensal microorganisms. This investigation revealed SL's ability to selectively target pathogenic oral bacteria while preserving commensal species as compared to conventional antiseptic rinses, CHX and LIS. Growth curves and viable bacterial counts in Figures 1, 2 demonstrated SL's ability to inhibit pathogenic bacterial proliferation while minimizing its impact on commensal microbes. Specifically, the growth profiles and CFU counts for *S. oralis*, *S. gordonii*, and *V. parvula* after treatment with SL were similar to that of PBS, demonstrating SL's minimal toxicity towards these commensal species. In contrast, *F. nucleatum* and *P. gingivalis* exhibited delayed growth profiles and reduced CFU counts up to 18 and 168 h, respectively, after SL treatment, which confirmed SL's antimicrobial activity against these gram-negative pathogens. Although CHX and LIS were more effective in inhibiting or eliminating *F. nucleatum* and *P. gingivalis* growth than SL, both rinses also severely stunted or eliminated commensal *S. oralis*, *S. gordonii*, and *V. parvula* growth. This lack of selective antimicrobial action is problematic as retention of health-related commensal bacteria is necessary to maintain oral mucosal immunity (29–31). Additionally, commensal bacteria contribute to averting infections caused by disease-associated opportunistic species (32).

Further corroborating these findings, these results show that SL sufficiently suppresses pathogens like *F. nucleatum* during multispecies bacterial regrowth after applied treatment and highlight the importance of preserving commensals after applied oral rinse treatment.

The findings of the present study align with a previous study, where CHX inhibited the growth of all bacterial species, including *S. mutans*, *S. sanguis*, *F. nucleatum*, and *P. gingivalis* when diluted to as low as 1.5% in growth medium whereas SL inhibited bacterial growth only at 25% (33). More importantly, SL was not cytotoxic to human gingival fibroblasts (HGF) and did not affect HGF proliferation at any dilution whereas CHX was cytotoxic at all dilutions (33). Similarly, Zhou et al. previously reported that SL was cytocompatible with cells critical to oral wound healing and promoted fibroblast migration and differentiation when compared to CHX (24).

To further validate the antimicrobial activity of SL, an *ex vivo* multispecies culture derived from a clinical periodontitis plaque sample was utilized per established protocol (26, 27). The multispecies culture was exposed to oral rinses in both planktonic (Figures 2A,B) and biofilm (Figures 4C,D) modes of growth. As observed for single species commensal cultures (Figure 1), SL did not impact the growth profile of planktonic multispecies cultures (Figure 4A) as compared to PBS while CHX and LIS reduced or eliminated cultivable bacterial growth, respectively. Interestingly, prolonged incubation (>1 week) of multispecies culture plating on agar resulted in the appearance of black-pigmented CFUs, typically associated with slower-growing, pathogenic anaerobes (34). Black-pigmented CFUs were present at ∼100,000 lower concentration than total CFUs prior to treatment (data now shown). As shown in Figure 4B, SL treatment resulted in fewer black-pigmented CFUs as compared to PBS control while CHX and LIS reduced or eliminated both black-pigmented CFUs. When comparing the impact of SL with CHX and LIS on black-pigmented CFU counts, SL is equally effective as LIS and CHX in reducing black-pigmented CFU counts after 48 h vs. PBS control.

*Ex vivo* multispecies culture formed a robust, microbially diverse biofilm as characterized by 16s rRNA sequencing confirming the presence of dozens of bacterial taxa (Supplementary Table S1) and corroborated by scanning electron microscopy (SEM) depicting various sizes of cocci, bacilli, and filamentous or rod-shaped bacteria (Supplementary Figure S1). As shown in Figure 4C, all oral rinses resulted in moderate bacterial cell death (red fluorescence) within biofilm as compared to PBS negative control which exhibited mostly live cells (green fluorescence) or IPA positive control which had the highest apparent number of dead cells. Corroborating this finding, semi-quantitative analysis (Figure 4D) of the amount of live (green) or dead (red) fluorescence signal revealed that naturopathic SL and conventional CHX or LIS resulted in significant decrease in live bacterial coverage to ∼40%–50% and significant increase in dead bacterial coverage to ∼50%–60% vs. PBS control (all *p* < 0.001). 3D reconstructions of the stained biofilms further confirmed a reduction in viable biofilm based on reduced biofilm thickness and smoother appearance. Thus, SL was as effective as CHX and LIS in neutralizing bacteria within the ecological *ex vivo* multispecies biofilm.

Cell spheroids derived from human gingival fibroblasts were used as a 3D model to assess cell viability following one-minute exposure to oral rinsing treatment as spheroids more accurately replicate the native tissue microenvironment. As shown in Figure 5, SL was the only treatment that maintained fibroblast spheroid viability and structure comparable to PBS-treated control. On the other hand, treatment by LIS or CHX resulted in substantial fibroblast death, with LIS resulting in moderate cell death within the outer shell of the spheroid and CHX penetrating and causing cell death within the spheroid core. These findings demonstrate the cytocompatibility of SL treatment as compared to CHX and LIS which induced pronounced cytotoxic effects in 3D fibroblast cultures.

Despite the findings of this study, several limitations need to be addressed. For assessment of selective antimicrobial activity against individual species, planktonic cultures were used to simulate treatment of bacteria immediately after biofilm disruption (e.g., mouthwash rinsing after brushing to remove dental plaque). While this method elucidated individual species susceptibility to oral rinses, oral bacteria exist as complex, multispecies biofilms with increased resistance to antimicrobial compounds (35). Future work will combine commensal and pathogenic species cultures into a multispecies biofilm model and study changes in biofilm composition after oral rinse treatment using quantitative PCR and selective agar plating. Secondly, an *ex vivo* multispecies biofilm was utilized to assess the efficacy of the tested oral rinses in eliminating biofilm. Although analysis was performed use live-dead staining, changes in the composition of the biofilm after oral rinse treatment were not evaluated. Subsequent studies will use 16S rRNA sequencing to assess changes in *ex vivo* multispecies biofilm composition after oral mouthwash treatment. Furthermore, the mechanism of action of the naturopathic rinse and its ability to selectively kill pathogenic gram-negative anaerobes will be investigated in future studies.

Overall, this study highlights the importance of formulating oral health treatments that specifically target pathogenic bacteria while preserving commensal microflora. It is imperative that mouthwashes used preventively do not eradicate the commensal microbiota of the periodontium, which has critical protective functions (2). In this context, the utilization of probiotics in oral health may hold great potential (36). Probiotics are living microorganisms that, when administered in adequate quantities, can confer a health benefit on the host (37). Future therapeutic methods may include probiotics combined with commensal-sparing mouthwashes to promote beneficial commensal bacteria in the oral microbiome (38). Introducing specific probiotic strains can help maintain a balanced oral microbiome, reverse dysbiosis, and support host modulation, thereby preventing conditions like periodontal disease and peri-implantitis (38, 39).

## 5 Conclusion

This study investigated the antibacterial and antibiofilm effects of StellaLife (SL), a novel homeopathic herbal mouthwash, compared to traditional rinses like chlorhexidine (CHX) and LISTERINE® (LIS). The findings highlight SL's potential to selectively inhibit pathogenic oral bacteria while preserving commensal microorganisms crucial for oral homeostasis. SL demonstrated significant antibacterial activity against periodontal pathogens *F. nucleatum* and *P. gingivalis*, showing efficacy similar to CHX and LIS. However, unlike these conventional rinses, SL exhibited minimal impact on beneficial commensals *S. oralis*, *S. gordonii*, and *V. parvula* that contribute to maintaining a balanced oral microbiome. Along with its previously reported cytocompatibility with human gingival fibroblasts and results from translational spheroid cultures of human fibroblasts presented in the results, SL may serve as a multifunctional alternative in oral care regimen and periodontal disease management by promoting wound healing while reducing pathogen load, thereby preserving oral microbial balance. Future research should focus on clinical trials to confirm these findings and explore the long-term benefits of combining probiotics with selective antimicrobial treatments. Overall, SL presents a promising step towards more targeted and sustainable oral healthcare strategies.

## Data Availability

The raw data supporting the conclusions of this article will be made available by the authors, without undue reservation.
