# Supplementary material for: Utilizing a naturopathic mouthwash with selective antimicrobial effects against multispecies oral biofilms for prevention of dysbiosis
Source: Front Oral Health. 2025 May 19;6:1529061. doi: 10.3389/froh.2025.1529061 (PMC12127372; doi:10.3389/froh.2025.1529061)
Supplement: Supplementary file 1 [file Datasheet1.docx]

Table S1. Top 50 most abundant bacterial taxa detected by 16S rRNA sequencing in *ex vivo* plaque-derived multispecies biofilm used in this study.

| **Genus** | **Species** |
| --- | --- |
| Fusobacterium | nucleatum_subsp._animalis |
| Peptostreptococcus | stomatis |
| Solobacterium | moorei |
| Fusobacterium | nucleatum_subsp._vincentii |
| Prevotella | buccae |
| Parvimonas | sp._HMT_110 |
| Porphyromonas | pasteri |
| Peptostreptococcaceae_[XI][G-1] | infirmum |
| Catonella | sp._HMT_164 |
| Prevotella | nigrescens |
| Rothia | dentocariosa |
| Rothia | aeria |
| Erythromicrobium | ramosum |
| Peptostreptococcus | anaerobius |
| Corynebacterium | matruchotii |
| Stomatobaculum | longum |
| Alloprevotella | rava |
| Capnocytophaga | sputigena |
| Actinomyces | sp._HMT_169 |
| Fusobacterium | nucleatum_subsp._polymorphum |
| Prevotella | sp._HMT_317 |
| Saccharibacteria_(TM7)_[G-1] | bacterium_HMT_952 |
| Atopobium | parvulum |
| Porphyromonas | sp._HMT_278 |
| Clostridiales_[F-3][G-1] | bacterium_HMT_876 |
| Porphyromonas | sp._HMT_275 |
| Bifidobacterium | dentium |
| Campylobacter | concisus |
| Capnocytophaga | leadbetteri |
| Alloprevotella | sp._HMT_473 |
| Leptotrichia | sp._HMT_215 |
| Lachnoanaerobaculum | orale |
| Campylobacter | gracilis |
| Mogibacterium | neglectum |
| Eggerthia | catenaformis |
| Tannerella | forsythia |
| Prevotella | oris |
| Sphingomonas | echinoides |
| Brevundimonas | diminuta |
| Agrobacterium | tumefaciens |
| Porphyromonas | sp._HMT_930 |
| Lachnospiraceae_[G-7] | bacterium_HMT_086 |
| Lachnospiraceae_[G-2] | bacterium_HMT_088 |
| Microbacterium | flavescens |
| Lachnospiraceae_[G-2] | bacterium_HMT_096 |
| Oribacterium | asaccharolyticum |
| Bacteroides | heparinolyticus |
| Pedobacter | sp._HMT_933 |
| Fastidiosipila | sanguinis |
| Capnocytophaga | sp._HMT_336 |

Table S2. Statistical analysis of the OD600 readings for each oral rinse as compared to PBS control within each timepoint and for each species tested.

|  |  |  |  |  |  |  |  |  |
| --- | --- | --- | --- | --- | --- | --- | --- | --- |
|  | ***S. oralis*** |  | **Growth Time Post-treatment (h)** | | | | |  |
|  |  | **Comparison** | 0 | 8 | 12 | 18 | 24 |  |
|  |  | PBS vs. CHX | ns | * | ns | * | * |  |
|  |  | PBS vs. LIS | ns | * | **** | **** | **** |  |
|  |  | PBS vs. SL | ns | * | ns | ns | ns |  |
|  |  |  |  |  |  |  |  |  |
|  | ***S. gordonii*** |  | **Growth Time Post-treatment (h)** | | | | |  |
|  |  | **Comparison** | 0 | 8 | 12 | 18 | 24 |  |
|  |  | PBS vs. CHX | ns | ns | ns | ns | **** |  |
|  |  | PBS vs. LIS | ns | ns | ns | ns | **** |  |
|  |  | PBS vs. SL | ns | ns | ns | ns | ns |  |
|  |  |  |  |  |  |  |  |  |
|  | ***V. parvula*** |  | **Growth Time Post-treatment (h)** | | | | |  |
|  |  | **Comparison** | 0 | 12 | 18 | 24 | 36 |  |
|  |  | PBS vs. CHX | ns | ns | ns | ns | **** |  |
|  |  | PBS vs. LIS | ns | ns | ns | ns | **** |  |
|  |  | PBS vs. SL | ns | ns | ns | ns | ns |  |
|  |  |  |  |  |  |  |  |  |
|  | ***F. nucleatum*** |  | **Growth Time Post-treatment (h)** | | | | |  |
|  |  | **Comparison** | 0 | 12 | 18 | 24 | 36 |  |
|  |  | PBS vs. CHX | ns | * | ** | *** | ns |  |
|  |  | PBS vs. LIS | ns | * | ** | ** | *** |  |
|  |  | PBS vs. SL | ns | * | *** | ns | ns |  |
|  |  |  |  |  |  |  |  |  |
|  | ***P. gingivalis*** |  | **Growth Time Post-treatment (h)** | | | | |  |
|  |  | **Comparison** | 0 | 48 | 96 | 120 | 168 |  |
|  |  | PBS vs. CHX | ns | ns | ns | ns | **** |  |
|  |  | PBS vs. LIS | ns | ns | ns | ns | **** |  |
|  |  | PBS vs. SL | ns | ns | ns | ns | **** |  |
|  |  |  |  |  |  |  |  |  |


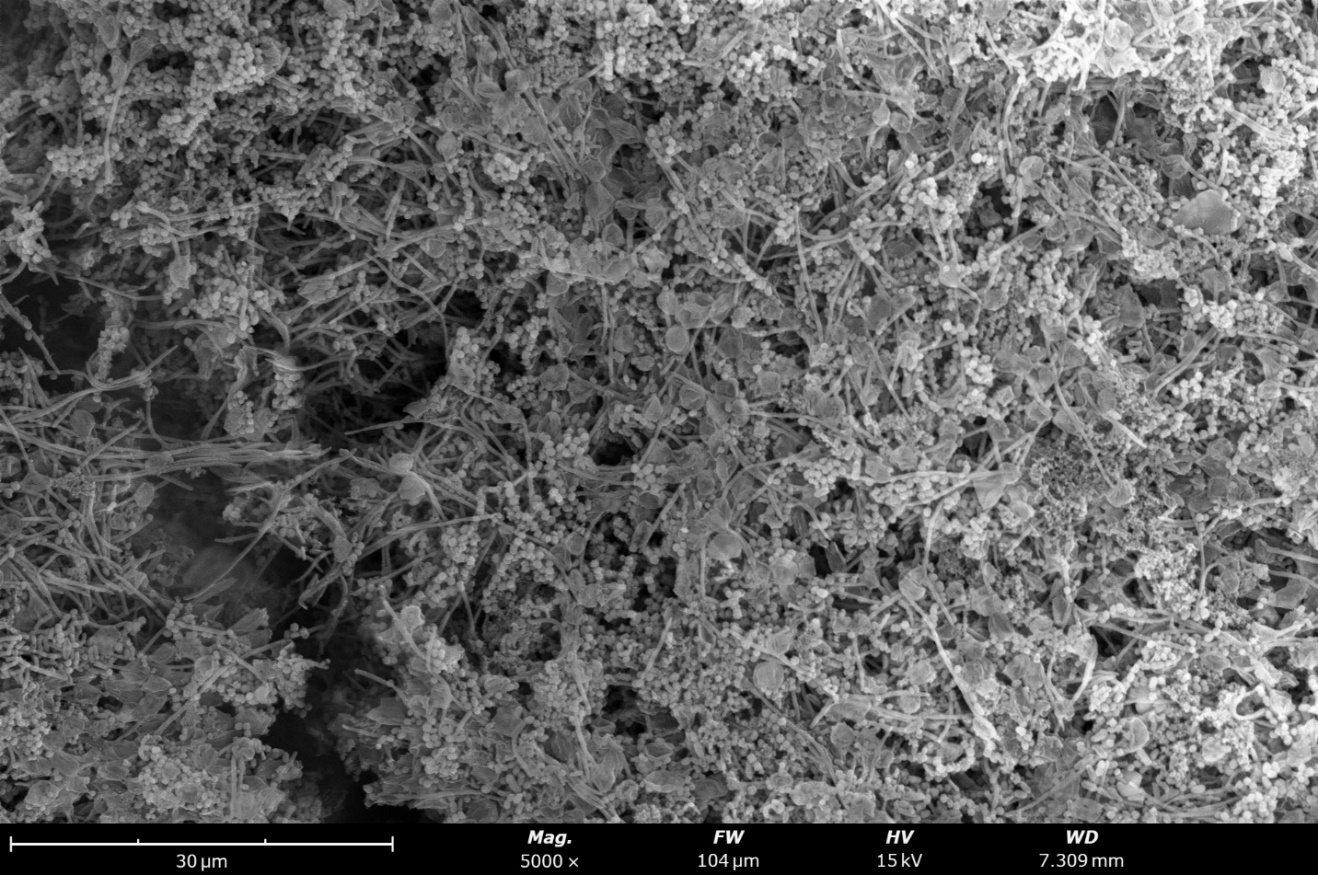


Figure S1. Scanning electron microscopy (SEM) image of *ex vivo* multispecies biofilm grown after 24 h.
